# Scaling A Positive Parenting Intervention from an Institutional Service to the Community Practice: A Qualitative Study of Net PAMA Classroom’s Success Factors and Challenges

**DOI:** 10.1192/j.eurpsy.2025.1121

**Published:** 2025-08-26

**Authors:** Q. Dejatiwongse Na Ayudhya, C. Pornnoppadol, S. Chanpen, W. Musikaphan

**Affiliations:** 1 Psychiatry, Siriraj Hospital, Mahidol University, Bangkok; 2 Institution for Population and Social Research, Mahidol University, Nakhonprathom, Thailand

## Abstract

**Introduction:**

Mental health issues in children and adolescents are often influenced by ineffective parenting strategies such as poor communication, harsh punishments, lack of positive reinforcement, and imbalance between relationship and regulation. Although effective parenting interventions exist, access is typically limited to institutional services. Net PAMA, an internet-based parent management training program, was created to improve access. However, only city-dwelling, educated parents were able to effectively learn from the platform. A combination of local facilitators and the internet-based program, called ‘Net PAMA Classroom’, was then proposed as means to reach out to underserved communities.

**Objectives:**

This study examines the success factors and challenges of ‘Net PAMA Classroom,’ a community-based Parent Management Training intervention conducted by non-professional facilitators, with the use of the internet-based standardized program.

**Methods:**

We interviewed fifteen Net PAMA Classroom facilitators. We then performed data triangulation and thematic analysis using Braun and Clarke’s six-step approach.

**Results:**

Three key themes were identified: curriculum, facilitators, and classroom management. Participants valued the practical skills provided through structured lessons. Successful facilitators had backgrounds in child and family work, prior facilitation experience, and supported parents in applying new skills. Support from community leaders significantly impacted the program’s initiation, delivery, and sustainability. Recommendations include making the curriculum culturally relevant, flexible, and less internet-dependent. Addressing propriety issues is also crucial for the program’s long-term success.

**Conclusions:**

The Net PAMA Classroom’s effectiveness relies on a robust curriculum, experienced facilitators, and effective classroom management. Enhancing cultural relevance and flexibility, reducing internet reliance, community support, and resolving propriety concerns are essential for sustainability and scalability.

**Disclosure of Interest:**

None Declared

